# Considerations for removal of cephalomedullary screws and blades following intertrochanteric femoral fracture healing: a narrative review

**DOI:** 10.1530/EOR-2026-0013

**Published:** 2026-06-01

**Authors:** Jee Young Lee, Gyu Min Kong

**Affiliations:** ^1^ Clinical Trial Center & Department of Microbiology, Gaspel Hospital, College of Medicine, Kosin University, Busan, Korea; ^2^ Department of Orthopaedic Surgery, Haeundae Paik Hospital, College of Medicine, Inje University, Busan, Korea

**Keywords:** cephalomedullary nail, femoral head collapse, fracture union, implant removal, intertrochanteric femoral fracture

## Abstract

**Background:**

- Cephalomedullary fixation using intramedullary nails combined with proximal lag screws or helical blades is widely accepted as the standard surgical treatment for unstable intertrochanteric femoral fractures. Although fracture union is reliably achieved in most patients, management of retained implants after healing remains controversial. Despite limited evidence supporting clinical benefit, elective implant removal continues to be performed in routine practice.

**Purpose:**

- This review synthesizes biomechanical, clinical, and health-economic evidence regarding the removal of cephalomedullary screws and helical blades after intertrochanteric fracture union. Particular emphasis is placed on mechanism-based structural failure, femoral head collapse, and age-dependent risk–benefit profiles.

**Methods:**

- A narrative literature review was conducted, incorporating biomechanical experiments, finite-element analyses, cadaveric studies, clinical case series, cohort studies, registry-based investigations, and health-economic analyses related to implant removal after intertrochanteric fracture fixation.

**Results:**

- Removal of proximal cephalic components produces a residual bone defect within the femoral head and neck, resulting in stress concentration and reduced structural integrity. Clinical studies report femoral neck fracture, varus collapse, and femoral head insufficiency, often occurring shortly after removal and frequently without significant trauma. These complications disproportionately affect elderly patients with osteoporotic bone. In contrast, symptomatic improvement after implant removal, particularly pain relief, is inconsistent and unpredictable.

**Conclusion:**

- Routine removal of cephalomedullary screws or helical blades after intertrochanteric fracture union is not supported by current evidence. Implant retention should be considered the default strategy, particularly in elderly patients, while selective removal may be considered only in carefully selected younger individuals with preserved bone quality and clear indications.

## Introduction

Intertrochanteric femoral fractures represent a major and growing global health burden, particularly in aging societies, and are associated with high morbidity, excess mortality, prolonged functional impairment, and substantial healthcare expenditure (1, 2, 3). As life expectancy increases worldwide, the incidence of intertrochanteric fractures is expected to rise further, amplifying the clinical and economic impact of their management.

Cephalomedullary fixation using intramedullary nails combined with proximal lag screws or helical blades has become the preferred surgical strategy for unstable intertrochanteric fractures (4, 5, 6, 7). Compared with extramedullary devices such as the dynamic hip screw, cephalomedullary systems offer biomechanical advantages, including a shorter lever arm, improved load-sharing characteristics, and reduced implant bending moments, resulting in high rates of fracture union across diverse patient populations (4, 5, 6, 7).

Despite reliable fracture healing, management of retained implants after union remains controversial. Indications for implant removal are often poorly defined, and outcomes following elective removal are unpredictable (8, 9). Nevertheless, elective removal continues to be performed in clinical practice, driven by patient expectations, surgeon habits, cultural beliefs, or theoretical concerns regarding pain, implant longevity, or long-term biological effects (8, 9, 10).

This issue is particularly complex for cephalomedullary systems, which incorporate large-diameter proximal components traversing the femoral head and neck. Removal of these components differs fundamentally from removal of plates or distal intramedullary nails and may result in predictable biomechanical weakening of the proximal femur (11, 12, 13, 14). Increasing reports of femoral neck fracture, varus collapse, and femoral head insufficiency after removal have raised concerns regarding the safety of routine implant removal, particularly in elderly patients with compromised bone quality (12, 15, 16, 17, 18).

Despite increasing clinical reports of post-removal mechanical failure, current practice remains largely guided by historical convention rather than mechanism-based evidence (8, 9, 10, 12, 15, 16, 17, 18). In particular, the widespread adoption of helical blade systems in aging populations has introduced new biomechanical considerations that are not adequately addressed in existing reviews. To date, no comprehensive synthesis has integrated biomechanical mechanisms, clinical failure patterns, and age-dependent risk stratification in the context of implant removal after intertrochanteric fracture union. Given the growing volume of relevant biomechanical data and accumulating reports of post-removal failure, a contemporary reassessment of this issue is timely.

## Methods

A narrative literature review was conducted using PubMed/MEDLINE, Embase, and the Cochrane Library. Searches focused on articles published between 1990 and 2025 to capture both foundational biomechanical investigations and contemporary clinical studies.

Search terms included combinations of intertrochanteric fracture, cephalomedullary nail, helical blade, lag screw, implant removal, femoral neck fracture, femoral head collapse, and refracture. Additional relevant studies were identified through manual screening of reference lists.

Biomechanical studies, finite element analyses (FEA), cadaveric experiments, clinical case reports, retrospective and prospective cohort studies, registry-based analyses, and health-economic evaluations were included. Given the heterogeneity of study designs and outcomes, evidence was synthesized qualitatively rather than through formal meta-analysis.

## Biomechanical consequences of cephalomedullary screw and blade removal

### Physiological load transmission and stress shielding

Under normal conditions, load transmission through the proximal femur depends on an organized trabecular architecture composed of primary compressive, primary tensile, and secondary trabecular systems. Cephalomedullary fixation alters this physiological load transmission by transferring a substantial portion of mechanical load from cancellous bone to the intramedullary nail and proximal cephalic component. While beneficial during fracture healing, this load-sharing mechanism may induce stress shielding and adaptive changes in bone architecture over time (14, 19).

Available imaging and biomechanical evidence are consistent with localized trabecular bone loss around the cephalic component after fracture union, particularly in osteoporotic bone (20, 21). These adaptive changes may not be fully reversible, especially in elderly patients with impaired osteogenic capacity.

### Residual bone defect and stress concentration

Removal of a lag screw or helical blade creates a substantial void within the femoral head and neck. This residual defect functions as a stress riser, concentrating mechanical loads in the surrounding cancellous bone and reducing overall structural integrity (11, 20, 22).

Biomechanical studies have demonstrated significant reductions in femoral head failure load following removal of proximal cephalic components. Reported reductions range from approximately 20% in normal bone to more than 50% in osteoporotic specimens (13, 23, 24, 25). These effects are particularly pronounced under non-axial loading conditions.

### Direction-dependent loading and failure

The biomechanical consequences of implant removal are strongly dependent on loading direction. While pure axial loading may be partially tolerated, varus bending and torsional stresses – common during activities such as stair climbing or rising from a chair – produce marked stress concentration around the residual defect (18, 22). This observation explains why many post-removal failures occur during routine daily activities rather than major trauma.

### Lag screw versus helical blade

Lag screws achieve fixation through thread purchase, leaving a threaded tract upon removal. Although this tract weakens local bone, some trabecular continuity remains between threads (26).

In contrast, helical blades rely on trabecular compaction rather than thread purchase. Removal disrupts compacted cancellous bone and creates a larger effective defect than lag screw removal (14, 27). Biomechanical studies suggest greater reductions in femoral head strength after blade removal, particularly under varus and torsional loading conditions (14, 22, 28).

**Figure 1:**
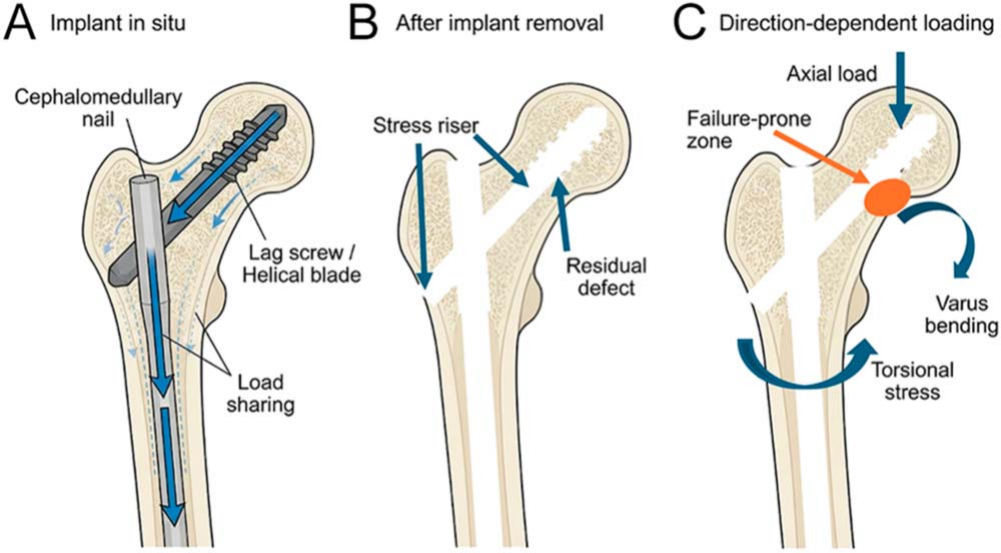
Biomechanical consequences of cephalomedullary implant removal in the proximal femur. (A) With the implant *in situ*, physiological load is partially transferred to the intramedullary nail and proximal cephalic component. (B) Implant removal creates a residual defect within the femoral head and neck, which acts as a stress riser. (C) Under non-axial loading, particularly varus bending and torsion, stress concentration around the residual defect increases the risk of mechanical failure.

These biomechanical mechanisms are summarized in Fig. 1, with representative supporting studies outlined in Supplementary Table S1 (see section on Supplementary materials given at the end of the article).

## Femoral head collapse as a distinct failure mechanism

Femoral head collapse following implant removal represents a distinct and under-recognized failure mechanism. Unlike an acute femoral neck fracture, collapse reflects progressive insufficiency of trabecular support within the femoral head and is most frequently reported after removal of helical blades (15, 16, 17, 18).

Removal of the blade eliminates the central compressive trabecular pillar of the femoral head. Under physiological loading, the superior portion of the femoral head may progressively sink into the residual cavity, producing varus drift of the femoral neck and increasing bending moments across the neck (16, 17, 18).

Although femoral head collapse may resemble avascular necrosis radiographically, the underlying cause is primarily mechanical rather than ischemic. Recognition of this distinction is critical for prevention and management.

## Implant-associated osteosarcoma: reassessment of risk

Concern regarding implant-associated osteosarcoma has historically been cited as a justification for routine implant removal. Early reports originated from outdated stainless-steel implants and galvanic corrosion (29). However, contemporary epidemiological studies in humans have not demonstrated a meaningful increase in osteosarcoma incidence associated with modern titanium or cobalt–chromium implants (30, 31, 32). Veterinary reports of implant-associated neoplasia – predominantly in dogs – should not be directly extrapolated to humans, given substantial interspecies differences in tumor biology and exposure contexts (33, 34). Accordingly, cancer prevention is not a valid indication for implant removal in contemporary orthopedic practice.

## Pain and functional outcomes after implant removal

Pain is the most commonly cited indication for elective implant removal (10, 35, 36, 37). However, persistent pain after intertrochanteric fracture healing is frequently multifactorial, including abductor dysfunction, iliotibial band irritation, heterotopic ossification, spinal pathology, and degenerative joint disease (35, 38).

Clinical studies demonstrate that pain relief after implant removal is inconsistent. Although some patients experience improvement, many report no change or worsening of symptoms, and a substantial proportion of previously asymptomatic patients develop new pain after removal (36, 37, 39). These findings underscore the limitations of pain alone as an indication for implant removal.

## Age-stratified risk–benefit profiles

### Younger patients

In younger patients with preserved bone quality, adaptive remodeling may partially compensate for the residual defect created by implant removal. Selective removal may be considered for clear mechanical symptoms, preparation for future reconstructive procedures, or strong patient preference after thorough counseling (13, 35, 40). Nevertheless, measurable reductions in femoral head strength still occur, and structural complications have been reported, including femoral neck fracture and collapse patterns (14, 15, 16, 17).

### Elderly patients

In elderly patients with osteoporotic bone, implant removal markedly increases the risk of femoral neck fracture, femoral head collapse, and early refracture (12, 15, 16, 17, 18, 41). Registry-based and population-level studies demonstrate that these complications often occur shortly after removal and may necessitate major revision surgery or conversion to arthroplasty (40, 41). For this population, implant retention represents the safest default strategy.

**Figure 2:**
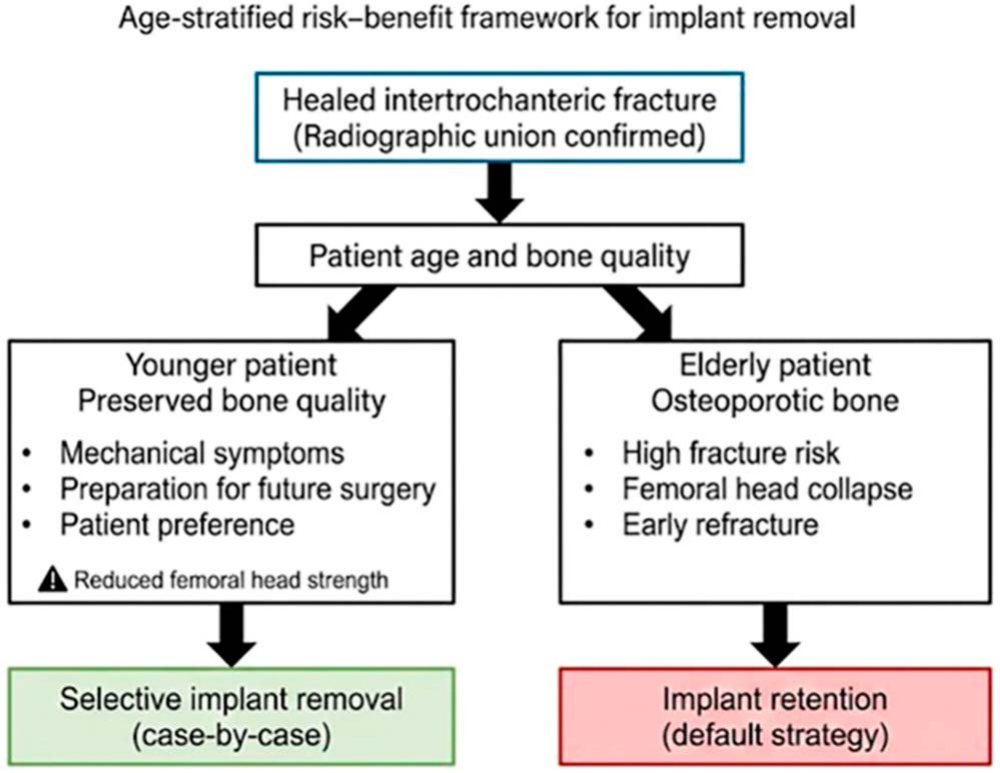
Age-stratified risk–benefit framework for cephalomedullary implant removal after fracture union. Clinical decision-making regarding implant removal should incorporate patient age, bone quality, and expected biomechanical risk. While selective implant removal may be considered in younger patients with preserved bone stock and clear indications, implant retention represents the safest default strategy in elderly patients with osteoporotic bone.

Based on the available biomechanical and clinical evidence, an age-stratified risk–benefit framework for cephalomedullary implant removal is proposed (Fig. 2).

## Health-economic considerations

Elective implant removal entails anesthesia exposure, hospitalization, postoperative rehabilitation, and indirect costs related to delayed functional recovery (42, 43). Health-economic analyses consistently question the cost-effectiveness of routine implant removal in the absence of clear clinical indications (42, 43, 44). When complications occur, costs escalate substantially, particularly if revision fixation or arthroplasty is required.

## Discussion

The management of retained implants following successful union of intertrochanteric femoral fractures remains an unresolved issue in contemporary orthopedic trauma practice. Although cephalomedullary fixation reliably achieves fracture healing, decisions regarding implant removal are characterized by substantial variability across institutions and surgeons, reflecting the absence of clear guidelines and the persistence of historical practice patterns (8, 9).

Routine implant removal after fracture union represents a legacy practice rooted in earlier generations of orthopedic implants, which were bulkier, less biocompatible, and more susceptible to corrosion or mechanical failure (27, 29). Although modern cephalomedullary systems are manufactured from highly biocompatible materials, the perception that retained metal constitutes incomplete treatment persists among both patients and surgeons (8, 35).

A central finding of this review is that complications following removal of cephalomedullary screws or helical blades are not stochastic events but predictable consequences of altered biomechanics. Removal of proximal cephalic components creates a residual bone defect that functions as a stress riser, concentrating mechanical loads in surrounding cancellous bone (11, 20). Finite element analyses and cadaveric studies consistently demonstrate significant reductions in femoral head failure load following removal, particularly in osteoporotic bone (13, 21, 23). These findings align with clinical observations that post-removal failures frequently occur under low-energy conditions rather than major trauma (12, 15, 39).

Femoral head collapse represents a distinct mechanical failure mode that differs fundamentally from acute femoral neck fracture. This mechanism is particularly relevant after helical blade removal, which disrupts compacted cancellous bone and eliminates the central compressive trabecular pillar of the femoral head (14, 15, 16, 17, 18). Although radiographic findings may resemble avascular necrosis, the temporal course and biomechanical context suggest a primarily mechanical etiology.

Pain-based indications for implant removal warrant careful scrutiny. Persistent pain after fracture healing is frequently multifactorial, and studies consistently demonstrate that pain relief after implant removal is unpredictable (35, 36, 37, 38, 39). Reliance on pain alone risks exposing patients to mechanical complications without a clear likelihood of symptomatic benefit.

Age functions as a critical surrogate for bone biology and risk. Younger patients may partially compensate for post-removal defects through adaptive remodeling, whereas elderly patients with osteoporotic bone lack this capacity and are at disproportionately high risk of mechanical failure (12, 38, 41). Registry-based data confirm higher rates of refracture and revision surgery in older populations following implant removal (41).

From a health-economic and ethical perspective, routine implant removal represents a questionable use of healthcare resources and exposes vulnerable patients to procedures with limited benefit and predictable risk (42, 43, 44). As value-based care becomes increasingly emphasized, avoidance of unnecessary implant removal aligns with responsible clinical practice.

### Future directions

Future research should focus on prospective registry-based studies to better quantify the incidence and predictors of mechanical failure following cephalomedullary implant removal, particularly in relation to patient age, bone quality, and implant design, as current evidence is largely derived from small case series and retrospective reports (12, 41). In addition, biomechanical and clinical investigations exploring prophylactic strategies – such as defect augmentation with bone graft or cement at the time of removal – may help mitigate post-removal structural vulnerability (11, 13). Finally, the development of mechanism-based clinical guidelines integrating biomechanical evidence with patient-specific risk stratification represents an important unmet need in contemporary fracture care.

## Conclusion

Routine removal of cephalomedullary screws or helical blades after intertrochanteric fracture union is not supported by current biomechanical, clinical, or economic evidence. Implant retention should be considered the default strategy, particularly in elderly patients with osteoporotic bone. Selective removal may be considered in younger patients with preserved bone quality and clear indications, but patients must be counseled that pain relief is uncertain and mechanical complications remain possible.

### Key messages

- Routine removal of cephalomedullary implants after fracture union lacks supportive evidence.
- Implant removal creates a predictable biomechanical vulnerability in the proximal femur.
- Femoral head collapse is a distinct mechanical failure mode, particularly after helical blade removal.
- Elderly patients with osteoporotic bone are at disproportionately high risk of post-removal failure.
- Implant retention should be the default strategy after uneventful fracture union.

## Supplementary materials



## ICMJE Statement of Interest

The authors declare that there is no conflict of interest that could be perceived as prejudicing the impartiality of the work reported.

## Funding Statement

This work did not receive any specific grant from any funding agency in the public, commercial, or not-for-profit sector.
