# Supplementary material for: Considerations for removal of cephalomedullary screws and blades following intertrochanteric femoral fracture healing: a narrative review
Source: EFORT Open Rev. 2026 Jun 1;11(6):591–7. doi: 10.1530/EOR-2026-0013 (PMC13261623; doi:10.1530/EOR-2026-0013)
Supplement: Supplementary file 1 [file supplementary_materials.pdf]

Supplementary Table S1. Summary of biomechanical and clinical evidence following cephalomedullary implant removal

Study	Study design	Model / Population	Loading condition or context	Key findings
Kukla et al. [13]	Cadaveric biomechanical study	Human cadaver proximal femur	Axial and bending loads	Removal of gamma nail significantly reduced femoral neck failure load compared with intact specimens
Mahaisavariya et al. [18]	Finite element analysis	Proximal femur model	Axial, varus bending, torsion	Marked stress concentration developed around the residual defect after nail removal, especially under non-axial loading
Hwang et al. [14]	Cadaveric biomechanical study	Human femoral head	Axial and rotational loads	Helical blade fixation altered trabecular load distribution; removal created a mechanically vulnerable region
Schwarz et al. [20]	Ex vivo biomechanical study	Human femur specimens	Multidirectional loading	Femoral fracture load decreased significantly after cephalomedullary nail removal, with greater reduction in osteoporotic bone
Yang et al. [24]	Biomechanical analysis	Proximal femur after implant	Axial and bending loads	Residual implant-related defects acted as stress risers and reduced

Study	Study design	Model / Population	Loading condition or context	Key findings
		removal		structural stiffness
Strauss et al. [23]	Cadaveric biomechanical study	Femoral neck defect model	Axial and bending loads	Cement augmentation partially restored femoral neck strength after implant removal
Barquet et al. [12]	Systematic clinical review	Patients with healed trochanteric fractures	Daily activities, low-energy trauma	Femoral neck fractures after implant removal frequently occurred during routine activities rather than major trauma
Yoon et al. [16]	Retrospective clinical study	Patients after CHS removal	Post-removal period	Increased risk of femoral neck fracture following implant removal in healed intertrochanteric fractures
Jin et al. [15]	Case report	Elderly osteoporotic patient	Post-removal ambulation	Early refracture occurred shortly after PFNA removal despite fracture union
Ponkilainen et al. [40]	Nationwide registry study	Finnish population database	Real-world clinical outcomes	Implant removal after proximal femur fracture was uncommon but associated with notable complication risk
